# Liposclerosing myxofibrous tumor of the distal femur: A case report

**DOI:** 10.3389/fsurg.2022.1009975

**Published:** 2023-01-06

**Authors:** Mingyang Zhang, Daguang Zhang, Wenjiang Yu, Chengxue Wang

**Affiliations:** Department of Orthopedics, The First Hospital of JilinUniversity, Changchun, China

**Keywords:** liposclerosing myxofibrous tumor, LSMFT, bone tumor, the distal femur, case report

## Abstract

**Introduction:**

Liposclerosing myxofibrous tumor (LSMFT) is a rare benign fibro-osseous tumor that most frequently occurs in the proximal femur. The reported literature shows that the proximal femur, ilium, tibia, humerus, rib, and skull have occurred, but so far, the female distal femur has not been characterized in detail. This, we think, is the first single comprehensive case report of the female distal femur. To prevent misdiagnosis and overtreatment of this illness, it is critical for us to continue strengthening our knowledge of it and to add it to the differential diagnosis of the space-occupying lesion of the female distal femur.

**Case summary:**

Two months ago, a 55-year-old female patient was found to have a space-occupying lesion of the left distal femur and the pain symptom was aggravated. She underwent thorough curettage and bone grafting without additional treatment to relieve the current symptoms and determine the nature of the lesion in our hospital. The intraoperative specimens were submitted to the pathology laboratory for analysis, and the result was reported as LSMFT. And six months after the operation, the patient returned to our hospital for another x-ray examination and we found that she had recovered well without any signs of recurrence. The patient self-reported that she had now resumed her daily life without any uncomfortable symptoms.

**Conclusion:**

The incidence of LSMFT itself is relatively low, and the occurrence of the distal femur is even rarer. However, it is recommended to add LSMFT into the differential diagnosis of the occupying lesions of the distal femur. Once the diagnosis is made, thorough curettage and bone grafting without additional special treatment can achieve better postoperative outcomes. The patient gave her agreement after learning that information about the case will be submitted for publication.

## Introduction

Liposclerosing myxofibrous tumor (LSMFT) was first described by Ragsdale and Sweet in 1986 (1). It is a relatively uncommon benign bone tumor that may arise in the proximal femur and other parts such as the ilium, humerus, tibia, ribs, and skull (1–5). The condition generally manifests itself around 40 years old, and the reported onset age is between 15 and 80 years old, with no notable gender difference (1–3, 6). The tumor is usually discovered by accident on radiology or with the painful symptom, and very rarely as a result of a pathological fracture (1).

In this rare case report, we document in detail a female patient who was found to have a space-occupying lesion in the distal femur and was diagnosed with LSMFT. She was treated only by complete curettage, and bone grafting and recovered satisfactorily. We think this may be the first case of LSMFT in the female distal femur that has been described in detail. We document the clinical, radiological, and pathological features as well as a retrospective discussion and summary of the case report.

## Case report

**Figure 1:**
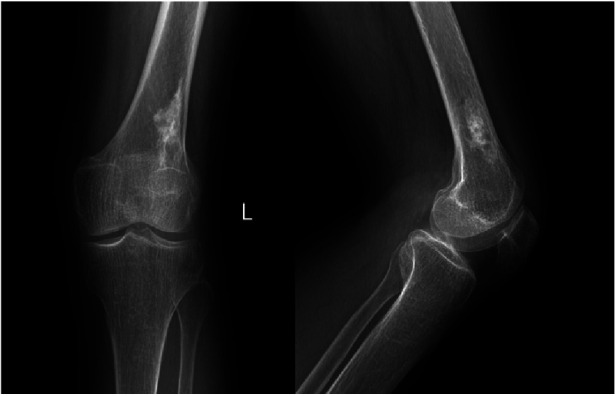
Anteroposterior and lateral radiographs of the left knee showing a patchy high-density shadow of the left distal femur without fracture or bone destruction.

**Figure 2:**
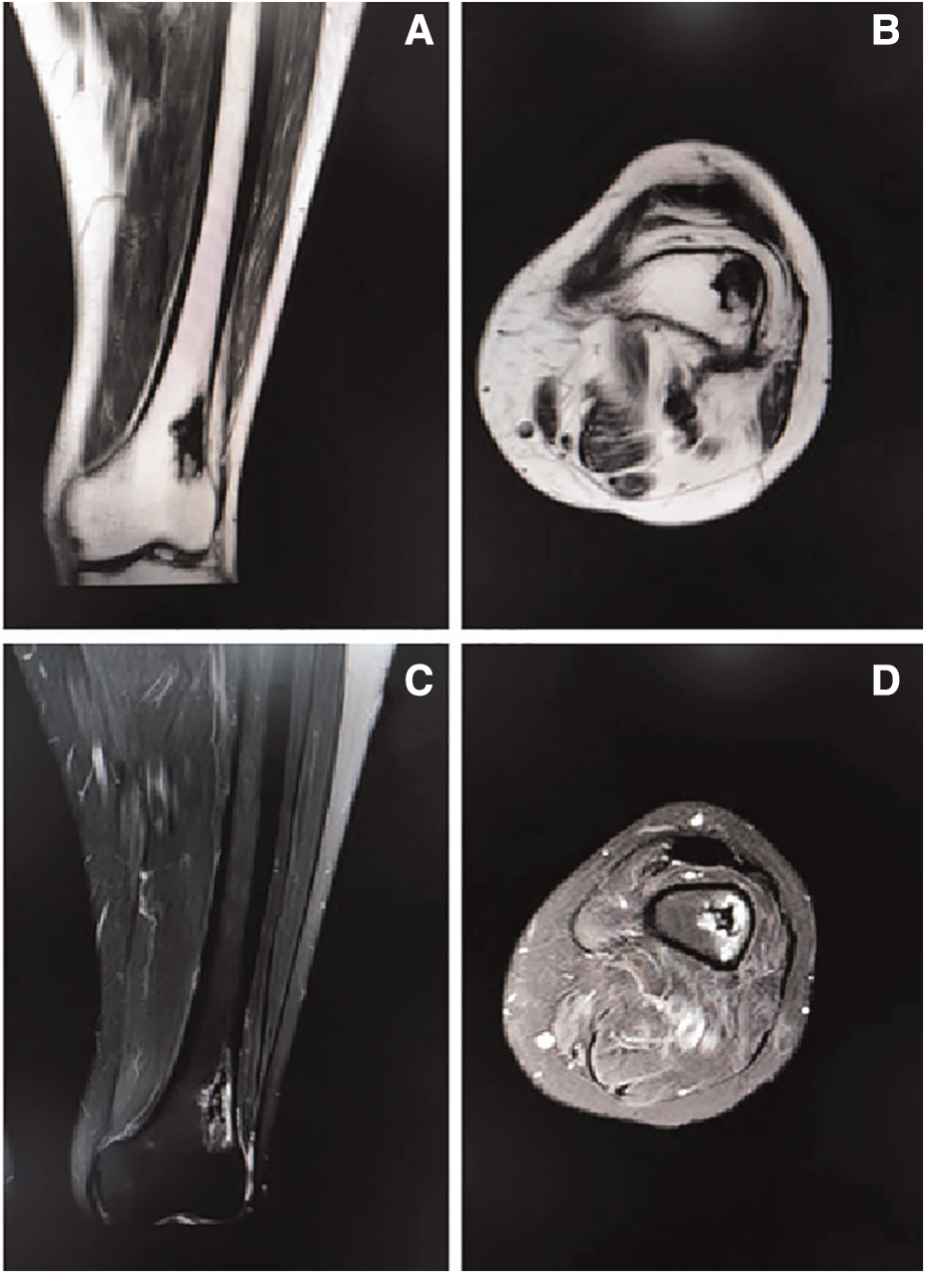
Representative coronal (Figure 2A) and axial (Figure 2B) T1-weighted images showing an isolated patchy abnormal signal of the left distal femur, with low signal intensity. Representative coronal (Figure 2C) and axial (Figure 2D) T2-weighted images showing a heterogenous lesion of the left distal femur with high signal intensity, with uneven internal signal, and no clear abnormal signal in the muscle and subcutaneous soft tissue of the left thigh.

**Figure 3:**
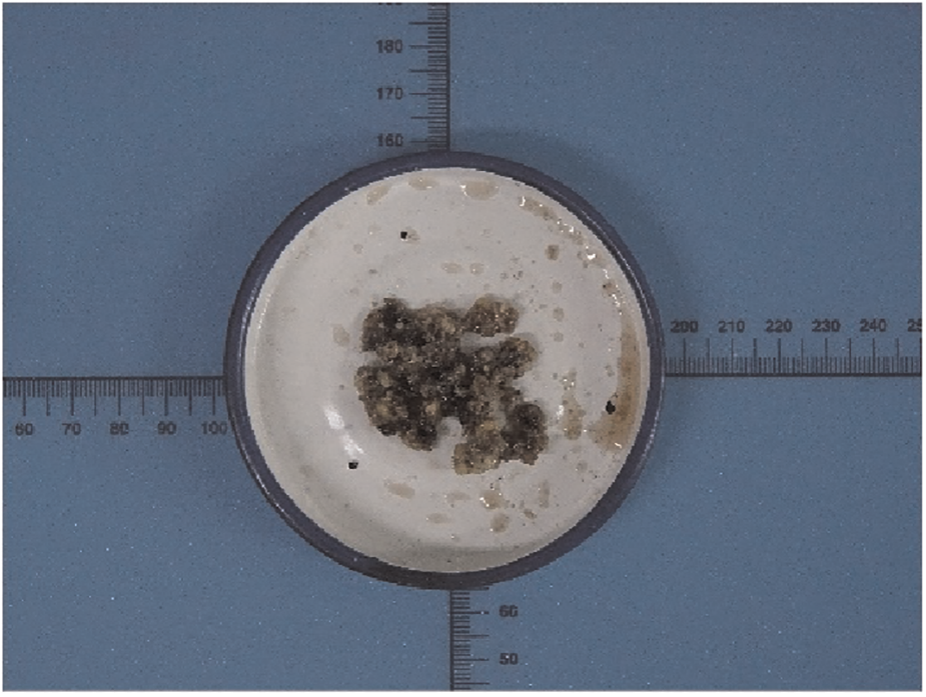
Histopathological examination of the specimens revealed that a pile of yellow broken bone tissues were visible to the naked eye.

**Figure 4:**
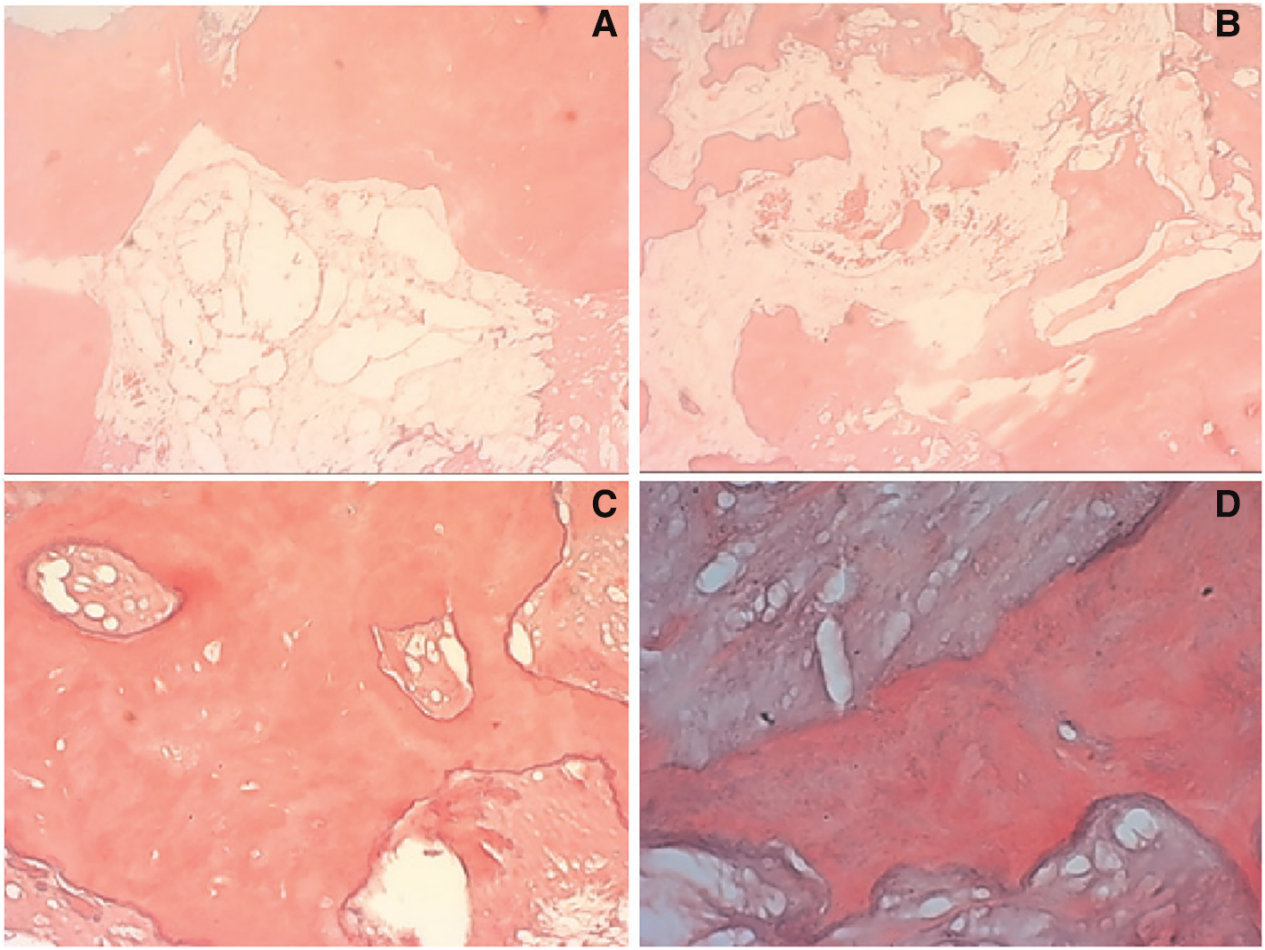
In some typical microscopic images, myxoid degeneration, calcified bone tissue, fat necrosis, cystic degeneration, irregular calcification, and collagen degeneration of fibrous tissue are seen (original magnifications of ×4 in (**A,B**, ×10 in (**C**), ×40 in **D**).

**Figure 5:**
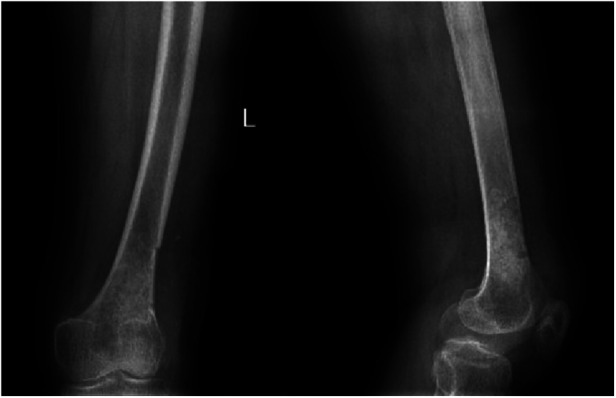
Anteroposterior and lateral radiographs of the left femur showing no abnormal signal at the left distal femur on the 3rd day after the surgery.

**Figure 6:**
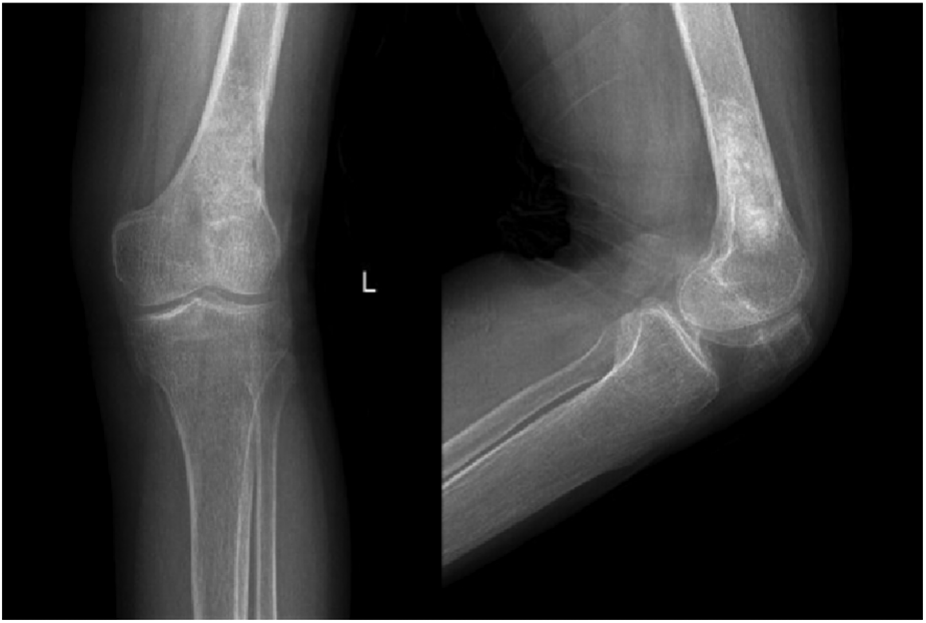
Follow-up anteroposterior and lateral radiographs of the left knee 6 months after the surgery showing good recovery of the left distal femur, consistent with postoperative changes of the left distal femur.

A 55-year-old female patient went to a local hospital for a magnetic resonance imaging (MRI) examination of her left knee 2 months ago because she felt swelling and dull pain in her left knee after daily walking. The result showed a space-occupying lesion in the left distal femur. The local doctor suggested conservative treatment and regular assessment. Conservative therapy lasted for one month, during which the symptoms did not improve, but the distension and pain of the left distal femur became more apparent, so she went to the orthopedic clinic in our hospital. She was found to have pain on the lateral side of her left distal femur by pressing, without touching the mass during the physical examination. The skin temperature of the left distal femur was normal and the skin could move freely. Anteroposterior and lateral radiographs of the left knee showed a patchy high-density shadow of the left distal femur without fracture or bone destruction (Figure 1). MRI of the left thigh showed an isolated patchy abnormal signal of the left distal femur, with low signal intensity on T1-weighted images (Figures 2A,B), as well as a heterogeneous lesion of the left distal femur with high signal intensity on T2-weighted images (Figures 2C,D), with uneven internal signal, and no clear abnormal signal in the muscle and subcutaneous soft tissue of the left thigh. We comprehensively considered it as a benign tumor initially and suspected it was endogenous chondrosarcoma. Nevertheless, there was no clear diagnostic foundation. In order to alleviate her current symptoms and ascertain the nature of the lesion, we admitted her to the hospital and began preparing her for surgery. Before the surgery, we carried out some examinations for her and found that all indicators of her hemoglobin, total white blood cell count and various specific indicators contained in white blood cell, platelets, coagulation function, calcium ions, potassium ions, antistreptolysin O, rheumatoid factor, hypersensitive c-reactive protein, fasting blood sugar, erythrocyte sedimentation rate, and renal function were normal. In addition to a slightly lower albumin index (37.3g/L, normal range: 40–55g/L) and a higher alkaline phosphatase index (174.8U/L, normal range: 50–135U/L), other indicators of hepatic function were normal. After a comprehensive analysis of preoperative examinations, no contraindications were found. We conducted surgical treatment for her on the third day in the hospital. An incision was performed on the lateral side of the left distal femur, and a long strip of the bone block with a size of 2 cm*8 cm was removed at the location of the tumor to expose the tumor. Additionally, the lesion in the medullary cavity was found to be fibrous-like and bone marrow-like in appearance. The lesion in the medullary cavity and on the bone block was thoroughly curetted until the normal bone was visible and all specimens were sent for histopathological analysis. The bleeding was thoroughly stopped, then the incision was rinsed with distilled water and normal saline. The artificial bone matrix was filled in the defect and the removed bone block is put in place. We did not perform plate fixation for her, which saved money for her, but increased the risk of postoperative fracture and the risk of thrombosis due to early failure of functional exercise. Histopathological examination of the specimens revealed that a pile of yellow broken bone tissues were visible to the naked eye, with a total volume of 4 cm*3 cm*1 cm (Figure 3). And in some typical microscopic images, myxoid degeneration, calcified bone tissue, fat necrosis, cystic degeneration, irregular calcification, and collagen degeneration of fibrous tissue are seen (Figure 4). LSMFT was confirmed after consulting with the pathology professors. We didn't give her follow-up radiation and chemotherapy because it's a benign lesion reported in the literature. The patient recovered well after the surgery. The alkaline phosphatase index decreased on the 1st day after the surgery and remained normal after the 3rd postoperative day. Anteroposterior and lateral radiographs of the left femur showed no aberrant signal at the left distal femur on the 3rd day after the surgery (Figure 5). On the fifth day after the operation, she was discharged from the hospital in good condition and returned home. During the routine follow-up evaluations for six months, she was found to have returned to normal daily life with no symptoms of discomfort. Six months later, anteroposterior and lateral radiographs of the left knee showed good recovery of the left distal femur, consistent with the postoperative change of the left distal femur (Figure 6). During the six months of follow-up, the symptoms of her affected limb showed no signs of recurrence, nor was there the indication of a recurrence of her imaging. We will continue to follow her up for a long period time and pay close attention to her life after surgery.

## Discussion

Liposclerosing myxofibrous tumor (LSMFT) was first described in 1986 and is most likely to occur in the proximal femur, accounting for 80%–90% of the cases (1, 2, 7). Some authors report that 48% of patients have pain at the site of onset, 40% are asymptomatic, and 10% have a pathological fracture (8). The age range of onset of this tumor is broad. According to the published literature, the age of onset is between 15 and 80 years old, the typical age is about 40 years old, and the incidence rate is equal between males and females (1). And this lesion is frequently a well-defined geographic lytic lesion with a sclerotic boundary (1). There is no conclusive explanation for the origin of the tumor. Some report that LSMFT is not a distinct lesion, but rather a traumatic variant of fibrous dysplasia or secondary degenerative changes of the lipoma (9–11). In addition, the Gs *α* mutation is also reported in two patients who are diagnosed with LSMFT, which is the similar mutation found in cases of fibrous dysplasia. Therefore, the authors of this paper believe that LSMFT is closely related to fibrous dysplasia (12). But some argue that the histological structure of LSMFT exceeds the range of histological structures currently reported for fibrous dysplasia and the tumor should therefore be treated as a distinct lesion (4, 7, 13, 14). Several authors even suggest that LSMFT should be classified as a true clinicopathological entity and included in textbooks of orthopedic pathology (14). It has been reported that the imaging manifestations of LSMFT are varied due to the complexity and diversity of histology. The imaging features of such lesion are usually the well-defined geographic lesion with extensive sclerosis of the margins, expansive remodeling, and mineralized matrix, which is inhomogeneous in density. Such features indicate an indolent pattern of growth. On T1-weighted images, the signal intensity of the lesion was comparable to that of skeletal muscle. On T2-weighted images, the signal intensity is greater than that of fat owing to the presence of myxoid tissue, which can be easily distinguished from intraosseous lipoma (3, 4, 15). The histological compositions of this tumor are complex and may consist of a variety of histological structures in varying proportions, including lipoma, fibroxanthoma, myxoma, myxofibroma, fibrous tissue, cyst formation, fat necrosis, ischemic bone tissue, and rarely cartilaginous tissue (1, 2, 4, 5, 7). Due to the diversity of histological forms, this tumor may easily be mistaken for fibrous dysplasia, intraosseous lipoma and other diseases (1, 16). Although LSMFT is a benign bone tumor, it has been reported to deteriorate into osteosarcoma, fibrosarcoma, telangiectatic osteosarcoma, malignant fibrous histiocytoma, malignant fibroxanthomas, and high-grade spindle cell tumor with a malignant transformation rate of 10% to 16%, which is significantly higher than the 0.5% malignant transformation rate of FD (4, 6, 7, 13, 16–18). The mechanisms of malignant transformation for LSMFT are unclear, but some authors suggest that the mechanisms are alterations in lipoma degeneration, changes in proliferative events in adipogenic lesions, and mechanical stress associated with ischemic damage (17, 19). The current consensus, however, is that LSMFT is a benign lesion, and its association with malignant transformation is controversial (3, 9, 15). According to the presently available literature, the asymptomatic patients with LSMFT usually do not require treatment and regular review is sufficient. If the patients have unpleasant symptoms such as pain, limping and pathological fracture, they can be treated by complete curettage, bone grafting, and orthopedic internal fixation, which depended on the specific situation. At the moment, the treatment of LSMFT is generally the surgical intervention without additional treatments such as radiation and chemotherapy, and the overall prognosis is wonderful. On the other hand, Regado et al. describe a patient with LSMFT who presented with pain and deformity and underwent transtibial amputation after the pathological biopsy with malignant cells. No recurrence or metastasis was found during the long-term follow-up (2). Campbell et al. described a patient with LSMFT who underwent the postoperative malignant transformation, tumor recurrence, and death in a short period after surgical treatments (6). The reports of malignant cases remind us to pay more attention to the postoperative recovery of the patients diagnosed with LSMFT.

## Conclusion

LSMFT has exhibited a considerable preference for the proximal femur. At the same time, ilium, humerus, ribs, and skull have also been reported. On the other hand, we have not found any literature detailing the occurrence of LSMFT in the female distal femur. This lesion often has a sclerotic border and is often a well-defined geographic lytic lesion. However, anteroposterior and lateral radiographs of the left knee showed a patchy high-density shadow of the left distal femur without fracture or bone destruction in this case. The diagnosis of this rare disease depends on a comprehensive analysis of clinical, radiological, and histopathological findings. The tumor may be readily mistaken for other lesions with possibly overlapping characteristics because of the diversity of its tissue structure. This rare case report is used to expand our awareness of LSMFT in the distal femur and to remind us to add LSMFT to the differential diagnosis before making a diagnosis of the space-occupying lesions of the female distal femur, with the final pathological diagnosis as the gold standard. Once the diagnosis is confirmed, thorough curettage and bone grafting are carried out without additional treatments. x-ray or MRI examinations are regularly ordered after the operation, and the patient's recovery status is closely followed. Generally, the postoperative recovery is excellent without affecting daily life.

## Data Availability

The original contributions presented in the study are included in the article/Supplementary Material, further inquiries can be directed to the corresponding author/s.

## References

[1] Deel C Hassell L. Liposclerosing myxofibrous tumor: a review. Arch Pathol Lab Med. (2016) 140(5):473–6. 10.5858/2014-0503-RS 27128305

[2] Regado ER Garcia PB Caruso AC de Almeida AL Aymoré IL Meohas W Liposclerosing myxofibrous tumor: a series of 9 cases and review of the literature. J Orthop. (2016) 13(3):136–9. 10.1016/j.jor.2016.03.003 27222619 PMC4821442

[3] Ploof J Shaikh H Melli J Jour G Turtz A. Liposclerosing myxofibrous tumor of the cranial vault: a case report. Neurosurg. (2019) 84(3):E207–10. 10.1093/neuros/nyy071 29538710

[4] Kransdorf MJ Murphey MD Sweet DE. Liposclerosing myxofibrous tumor: a radiologic-pathologic-distinct fibro-osseous lesion of bone with a marked predilection for the intertrochanteric region of the femur. Radiol. (1999) 212(3):693–8. 10.1148/radiology.212.3.r99se40693 10478234

[5] Choi JW Lee YS Lee JH Kim HK Yeom BW Choi JS Liposclerosing Myxofibrous Tumor in Tibia: A Case Report and Review of the Literature. 2005.

[6] Campbell K Wodajo F. Case report: two-step malignant transformation of a liposclerosing myxofibrous tumor of bone. Clin Orthop Relat Res. (2008) 466(11):2873–7. 10.1007/s11999-008-0362-9 18607664 PMC2565027

[7] Ragsdale BD. Polymorphic fibro-osseous lesions of bone: an almost site-specific diagnostic problem of the proximal femur. Human Pathl. (1993) 24(5):505–12. 10.1016/0046-8177(93)90162-A 8491490

[8] Tecualt-Gomez R Atencio-Chan A Cario-Mendez AG Amaya-Zepeda RA Balderas-Martinez J Gonzalez-Valladares JR. Bone liposclerosing myxofibrous tumor. Case presentation and literature review. Acta Ortop Mex. (2015) 29(3):191–5. PMID: 26999973

[9] Dattilo J McCarthy EF. Liposclerosing myxofibrous tumor (LSMFT), a study of 33 patients: should it be a distinct entity? Iowa Orthop J. (2012) 32:35–9. PMID: 23576919 PMC3565412

[10] Corsi A De Maio F Ippolito E Cherman N Gehron Robey P Riminucci M Monostotic fibrous dysplasia of the proximal femur and liposclerosing myxofibrous tumor: which one is which? J Bone Miner Res. (2006) 21(12):1955–8. 10.1359/jbmr.060818 17002568

[11] Heim-Hall JM Williams RP. Liposclerosing myxofibrous tumour: a traumatized variant of fibrous dysplasia? Report of four cases and review of the literature. Histopathol. (2004) 45(4):369–76. 10.1111/j.1365-2559.2004.01951.x 15469475

[12] Matsuba A Ogose A Tokunaga K Kawashima H Hotta T Urakawa S Activating gs alpha mutation at the Arg201 codon in liposclerosing myxofibrous tumor. Hum Pathol. (2003) 34(11):1204–9. 10.1016/S0046-8177(03)00430-1 14652823

[13] Beytemür O Tetikkurt Ü S Albay C Kavşut G Güleç A. Liposclerosing myxofibrous tumor: a rare tumor of proximal femur. Eklem Hastalik Cerrahisi. (2017) 28(3):210–3. 10.5606/ehc.2017.48394 29125822

[14] Gilkey FW. Liposclerosing myxofibrous tumor of bone. Hum Pathol. (1993) 24(11):1264. 10.1016/0046-8177(93)90226-7 8244329

[15] O’Dwyer H Al-Nakshabandi NA Saliken J Munk PL Nielsen TO Masri B Liposclerosing myxofibrous tumour. Eur. J. Radiol. 2005, 55 (3), 83–7. 10.1016/j.ejrex.2005.07.009

[16] Kheok SW Ong KO. Benign periarticular, bone and joint lipomatous lesions. Singapore Med J. (2017) 58(9):521–7. 10.11622/smedj.2017087 28948289 PMC5605823

[17] Bahk WJ Seo KJ. Malignant transformation of liposclerosing myxofibrous tumour. Pathol. (2021) 53(5):660–3. 10.1016/j.pathol.2020.09.032 33745703

[18] Illac C Delisle MB Bonnevialle P Chiavassa-Gandois H de Pinieux G Gomez-Brouchet A. [Telangiectatic osteosarcoma secondary to a liposclerosing myxofibrous tumor: a case report]. Ann Pathol. (2012) 32(4):259–62. 10.1016/j.annpat.2012.06.003 23010399

[19] Murphey MD Carroll JF Flemming DJ Pope TL Gannon FH Kransdorf MJ. From the archives of the AFIP: benign musculoskeletal lipomatous lesions. Radiographics. (2004) 24(5):1433–66. 10.1148/rg.245045120 15371618

